# Multiple TLRs elicit alternative NLRP3 inflammasome activation in primary human monocytes independent of RIPK1 kinase activity

**DOI:** 10.3389/fimmu.2023.1092799

**Published:** 2023-10-26

**Authors:** Sarah Unterberger, Lisa Mullen, Melanie S. Flint, Sandra Sacre

**Affiliations:** ^1^ Brighton and Sussex Medical School, University of Sussex, Brighton, United Kingdom; ^2^ Centre for Stress and Age-related Disease, School of Applied Sciences, University of Brighton, Brighton, United Kingdom

**Keywords:** interleukin-1β (IL-1β), toll-like receptors (TLRs), NLRP3 inflammasome, primary human monocytes, caspase-8, gasdermin D

## Abstract

The canonical NOD-like receptor family pyrin domain containing 3 (NLRP3) pathway involves a priming step to induce pro-IL-1β followed by a secondary signal such as K^+^ efflux to activate inflammasome formation. This then leads to the maturation of IL-1β and the formation of gasdermin D (GSDMD) pores that initiate pyroptosis and mediate IL-1β release. In contrast, primary human monocytes also engage an alternative pathway in response to toll-like receptor (TLR) 4 activation, without the need for a secondary signal. Data from a monocyte-like cell line suggest that the alternative pathway functions via the TLR adaptor protein TIR-domain-containing adapter-inducing interferon-β (TRIF), receptor-interacting protein kinase 1 (RIPK1), FAS-associated death domain (FADD) and caspase-8 upstream of NLRP3 activation, but in the absence of K^+^ efflux or pyroptosis. Usage of the alternative pathway by other members of the TLR family that induce IL-1β but do not signal through TRIF, has yet to be explored in primary human monocytes. Furthermore, the mechanism by which IL-1β is released from monocytes remains unclear. Therefore, this study investigated if the alternative NLRP3 inflammasome pathway is initiated following activation of TLRs other than TLR4, and if GSDMD was necessary for the release of IL-1β. Monocytes were stimulated with ligands that activate TLR1/2, TLR2/6, TLR4 and TLR7 and/or TLR8 (using a dual ligand). Similar to TLR4, all of the TLRs investigated induced IL-1β release in a NLRP3 and caspase-1 dependent manner, indicating that TRIF may not be an essential upstream component of the alternative pathway. Furthermore, inhibition of RIPK1 kinase activity had no effect on IL-1β release. Although IL-1β was released independently of K^+^ efflux and pyroptosis, it was significantly reduced by an inhibitor of GSDMD. Therefore, it is feasible that low level GSDMD pore formation may facilitate the release of IL-1β from the cell, but not be present in sufficient quantities to initiate pyroptosis. Together these data suggest that the alternative pathway operates independently of RIPK1 kinase activity, downstream of diverse TLRs including TLR4 in primary human monocytes and supports the potential for IL-1β release via GSDMD pores alongside other unconventional secretory pathways.

## 1 Introduction

Interleukin (IL)-1β is a key inflammatory cytokine that has a broad activity causing fever, inducing acute-phase proteins, and recruiting and activating immune cells (1). IL-1β can be released in response to infection but has also been associated with the pathogenesis of many sterile inflammatory diseases, including gout, type 2 diabetes and rheumatoid arthritis (1–3). In response to cellular activation, IL-1β is produced as a pro-form that requires processing by a multiprotein complex, known as the inflammasome (4). There are several different sensors that can form an inflammasome of which the NOD-like receptor family pyrin domain containing 3 (NLRP3) is the most widely studied (5).

In most cell types, activation of the NLRP3 inflammasome follows a two signal-model termed the canonical NLRP3 pathway (6). First, a priming signal occurs, which can be activated by stimulation of pattern recognition receptors or cytokine receptors to induce the transcription of *NLRP3* and *IL1B* through activation of nuclear factor-κB (NF-κB) (7). A second signal then activates NLRP3 which oligomerises with the adaptor apoptosis-associated speck-like protein containing a CARD (ASC) to form an activation platform resulting in the recruitment of pro-caspase-1 (8). This second signal can be initiated by various stimuli including microbial products, such as bacterial toxins, or endogenous molecules like ATP, which induce K^+^ efflux (9, 10). Once the inflammasome is formed, pro-caspase-1 is cleaved leading to its activation and in turn, caspase-1 cleaves pro-IL-1β (to a 17kDa mature cytokine) and gasdermin D (GSDMD) (4, 11). The N-terminal fragments of GSDMD oligomerise forming pores in the membrane permitting the release of IL-1β, which lacks a signal sequence to direct protein export via the endoplasmic reticulum and Golgi apparatus (12, 13). These pores additionally trigger pyroptosis, an inflammatory cell death characterised by osmotic cell lysis (11, 14, 15).

The NLRP3 inflammasome can also be activated by the non-canonical pathway when cytosolic lipopolysaccharide (LPS) is sensed by caspase-11 in mice and caspase-4/5 in human cells (16, 17). This initiates GSDMD cleavage causing K^+^ efflux which promotes NLRP3 inflammasome assembly (18). However, in primary human monocytes, a further pathway has been identified, termed the alternative pathway, whereby IL-1β is released in response to toll-like receptor (TLR)4 activation without the need for a second signal and independent of caspase-4. This pathway was shown to engage TIR-domain-containing adapter-inducing interferon-β (TRIF), receptor-interacting protein kinase 1 (RIPK1), FAS-associated death domain (FADD) and caspase-8 upstream of NLRP3 and caspase-1, but was independent of K^+^ efflux and pyroptosis (19). Consequently, due to an absence of pyroptosis, the mechanism by which IL-1β is released from human monocytes via the alternative pathway remains unclear. Furthermore, as TRIF is an adaptor for TLR3 and TLR4 but not the other members of the TLR family that are known to induce IL-1β release from human monocytes, it is not known if other TLRs can activate this pathway (20). Thus, this study set out to investigate if the alternative pathway is engaged by TLRs that do not signal through TRIF in primary human monocytes and to explore if GSDMD retains a role in IL-1β release in the absence of pyroptosis.

## 2 Materials and methods

### 2.1 Isolation and cell culture of primary human monocytes

Healthy donor blood was purchased from the National Health Service Blood and Transplant (NHSBT) (Tooting, UK). Written informed consent was obtained by NHSBT. Ethical approval for the use of human blood purchased from the NHSBT was granted by Wales research ethics committee 6 (18/WA/0176). Peripheral blood mononuclear cells (PBMCs) were isolated using Ficoll-Paque gradients (Cedarlane, Burlington, Canada) as previously described (21). Peripheral blood monocytes were isolated from PBMCs by iso-osmotic Percoll gradient centrifugation as previously described (22). Monocytes were cultured at 37°C and 5% CO_2_ for 4-24 hours. Cells were cultured in RPMI 1640 media containing 5% (v/v) FBS and 100U/ml penicillin/streptomycin or Opti-MEM containing 100U/ml penicillin/streptomycin.

Cells were stimulated with 100ng/ml PAM3CSK4 (Pam3) (Axxora, Nottingham, UK), 10ng/ml LPS (Axxora), 1ng/ml Pam2CGDPKHPKSF (FSL-1) (Invivogen, San Diego, CA, USA), or 2µg/ml Resiquimod (R-848) (Enzo Life Sciences, Lausen, Switzerland) in the presence or absence of 10µM MCC950 (gifted by Dr. Matthew Cooper from University of Queensland, Australia), 10µM nigericin (Invivogen), 20mM potassium chloride (KCl) (Carl Roth, Karlsruhe, Germany), 40μM Necrostatin-1 (Nec-1) (Thermo Fisher Scientific, Waltham, MA USA), 2.5μM GSK872 (Biotechne, Abingdon, UK) or 10µM necrosulfonamide (NSA) (Biovision, CA, USA). For caspase inhibition, cells were pre-incubated for 30 min with 10µM Z-YVAD-FMK (caspase-1 inhibitor) (Calbiochem, Millipore, Hertfordshire, UK) or 1µM Z-IETD-FMK (caspase-8 inhibitor) (Calbiochem) before stimulation. Staurosporine (1µM) (Calbiochem) was used to induce caspase-8 activation. Cell viability was assessed using the CellTiter-blue Cell Viability Assay (Promega, Madison, WI, USA) according to the manufacturer’s instructions. All experiments were performed as technical triplicates for each donor from which the mean is displayed in graphs where the data has been pooled from several donors.

### 2.2 Lactate Dehydrogenase (LDH) cytotoxicity assay

The Pierce LDH Cytoxicity Assay Kit (Thermo Fisher Scientific, Waltham, MA USA) was used to quantify pyroptosis according to manufacturer’s instructions. To determine LDH activity, the 680nm absorbance value was subtracted from the 490 absorbance. Relative LDH release was calculated as LDH release [%] = (compound treated LDH activity – spontaneous LDH activity)/(maximum LDH activity – spontaneous LDH activity) x 100.

### 2.3 Caspase activity assays

Caspase-1 and caspase-8 activity were measured in cell lysates and supernatants using Caspase-Glo 1 inflammasome assay and Caspase-Glo 8 assay (Promega) according to manufacturer’s instructions.

### 2.4 ELISA

Sandwich ELISAs were applied to measure IL-1β and tumor necrosis factor-α (TNF) in cell supernatants. IL-1β capture antibody (MAB601, R&D systems, Abingdon, UK), and biotinylated IL-1β antibody (BAF201, R&D systems), TNF capture antibody (551220, BD Pharmingen, UK), TNF biotinylated antibody (554511, BD Pharmingen) were used according to the manufacturer’s instructions. Recombinant TNF and IL-1β were purchased from PeproTech (London, UK). Streptavidin horseradish peroxidase (DY998, R&D systems) and 3,3′,5,5′-Tetramethylbenzidine microwell peroxidase substrate kit (KPL Inc., USA) were used according to the manufacturer’s instructions. Colour formation was stopped using 0.16 M sulfuric acid. Absorbance was read at 450 nm on a Biotek synergy HT Microplate reader and analysed using Gen5 software (BioTek, Winooski, VT, USA). The IL-1β ELISA detects both mature and pro-IL-1β.

### 2.5 Quantitative polymerase chain reaction

RNA was extracted using a RNeasy Mini Kit (QIAGEN, Stockach, Germany) and reverse transcribed using a High Capacity cDNA Reverse Transcription kit (Applied Biosystems, Paisley, UK) according to the manufacturer’s instructions. The cDNA was analysed using Taqman qPCR assays (Life Technologies, Carlsbad, USA) using Taqman PCR master mix 2x (Thermo Fisher Scientific, Waltham, MA, USA). The expression of *IL1B* (assay: Hs00174097_m1) *and NLRP3* (Hs00918082_m1) were determined relative to the geometric mean of the reference genes *GAPDH* (Hs02758991_g1) and *HPRT1* (Hs02800695_m1). Reactions were performed using an Agilent AriaMX thermocycler (Agilent Technologies, Cheshire, UK) as per manufacturer’s instructions. Agilent Aria 1.6 software (Agilent Technologies) was used for data collection and analysis. The comparative threshold cycle method (2^-ΔCt^) was used for quantification of gene expression.

### 2.6 Western blot

Cells were lysed in Triton X buffer [1% Triton-X (Sigma-Aldrich], 2mM EDTA (Fisher Scientific, Loughborough, UK), 100mM NaCl (Fisher), 30mM HEPES pH7.5 (Melford, Ipswich, UK)] or NP-40 buffer [50mM TrisHCl pH8 (Sigma-Aldrich), 150mM NaCl (Fisher), 1% IGEPAL-CA-630 (Sigma-Aldrich)] containing freshly added protease inhibitor (Sigma-Aldrich). Protein concentrations were determined using a bicinchoninic acid protein assay kit (Thermo Fisher Scientific) according to the manufacturer’s instructions. Proteins in the supernatants were precipitated in acetone at -20°C overnight, clarified at 12000xg for 10min and then resuspended in 2X SDS-PAGE loading buffer. Lysate-proteins were denatured in 5X SDS-PAGE loading buffer, separated on tris-glycine denaturing SDS-PAGE and transferred onto nitrocellulose membranes (GE Healthcare Life Sciences, Little Chalfont, UK). After transfer, total protein staining was performed using Revert™ 700 Total Protein Stain (LI-COR, Lincoln, NE, USA) according to the manufacturer’s instructions. Membranes were blocked with 5% milk in Tris-buffered saline with 0.1% Tween 20 (TBS-T) for 1h at RT and incubated overnight at 4°C with anti-IL-1β (MAB601, R&D). For GSDMD detection, membranes were blocked and incubated with anti-GSDMDC1 Antibody (64-Y) (sc-81868, Santa Cruz Biotechnology) in intercept blocking buffer (LI-COR) in TBS-T. Membranes were then incubated with m-IgGκ BP-CFL 790 Antibody (sc-516181, Santa Cruz Biotechnology) in 5% milk in TBS-T. For protein visualisation, fluorescence was measured on a LI-COR Odyssey Fc (LI-COR).

### 2.7 Statistical tests

Mean, standard deviation, standard error of the mean (SEM), and statistical significance were calculated using GraphPad version 8 (GraphPad Software Inc., USA). For statistical analysis, parametric data were compared using a two-tailed one sample t-test. Non-parametric data were compared with a two-tailed one sample Wilcoxon test. For the comparison of multiple data sets relative to a control, one-way ANOVA with posthoc Dunnett’s multiple comparisons test was applied for parametric distributed data, in case of a non-parametric distribution, Kruskal-Wallis test with posthoc Dunnett’s multiple comparisons test was used. In case of multiple comparisons of non-parametric distributed datasets, a Friedman test was used. Significance is shown as *p ≤ 0.05, **p ≤ 0.01, ***p ≤ 0.001.

## 3 Results

### 3.1 Primary human monocytes do not require a second signal to release IL-1β upon TLR stimulation

**Figure 1:**
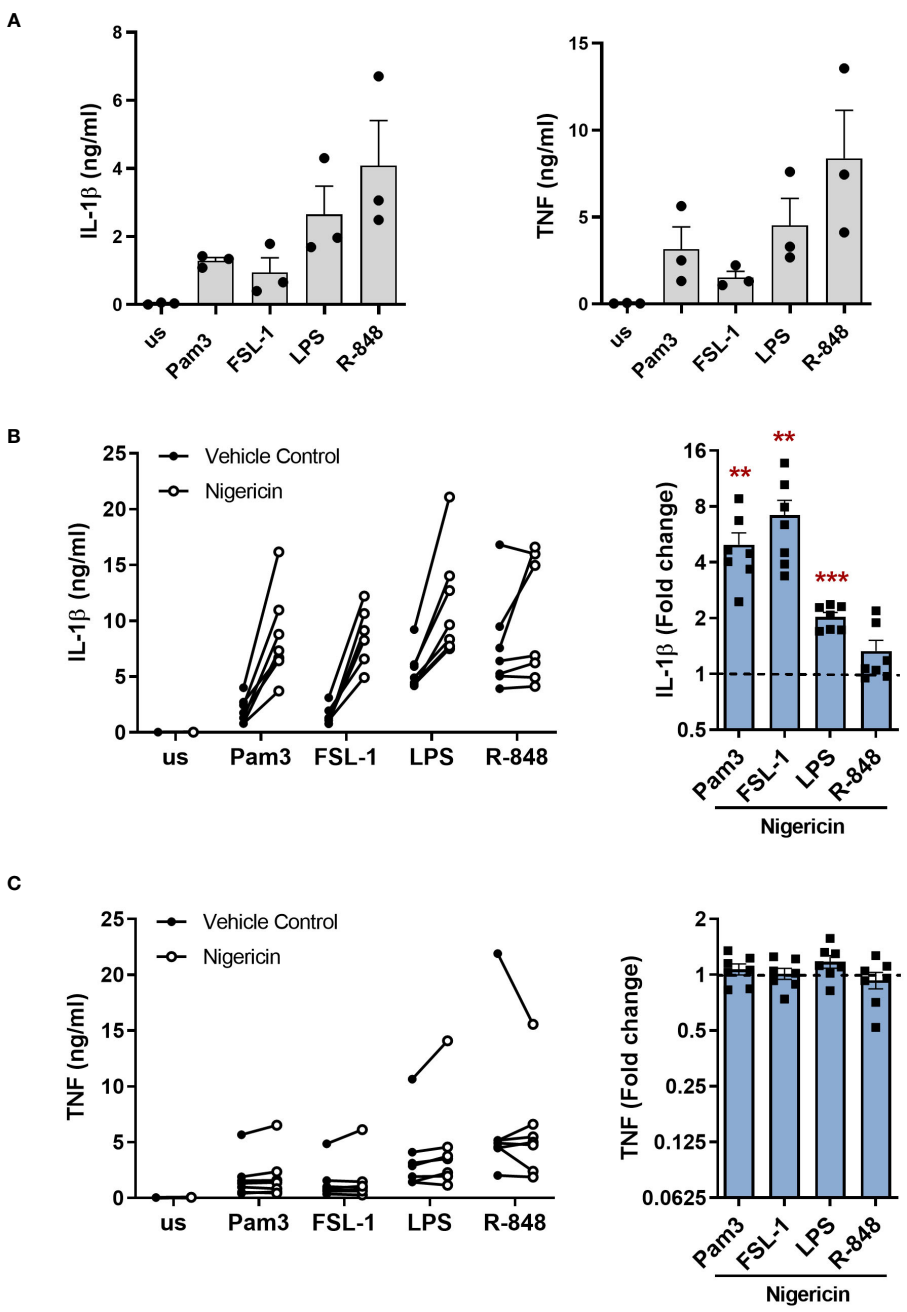
Activation of TLRs induces IL-1β release in primary human monocytes which is amplified by nigericin. **(A-C)** Primary human monocytes were unstimulated (us) or stimulated for 24h with 100ng/ml Pam3, 1ng/ml FSL-1, 10ng/ml LPS or 2µg/ml R-848. IL-1β and TNF secretion were measured. **(A)** Data are shown as the mean ± SEM from 3 individual donors. **(B, C)** Cells were additionally incubated with 10µM nigericin during the last 2h of stimulation. Data are displayed as the mean of technical triplicates (left) and as the fold change normalised to the corresponding TLR activation without nigericin (dotted line) and pooled as the mean ± SEM (right) from 7 individual donors. Significance was determined using two-tailed one sample t-test or one sample Wilcoxon test against the response without nigericin treatment (**p ≤ 0.01, ***p ≤ 0.001).

To investigate if the alternative NLRP3 inflammasome pathway is activated by different TLRs in primary human monocytes, cells were stimulated for 24h with Pam3 (TLR1/2), FSL-1 (TLR2/6), LPS (TLR4) and R-848 that can stimulate both TLR7 and TLR8. All TLR ligands induced IL-1β and TNF secretion (**Figure 1A**). However, addition of the K^+^ ionophore nigericin during the last 2h of stimulation to activate the canonical NLRP3 pathway, as would occur in the canonical model of NLRP3 inflammasome activation, significantly increased the level of IL-1β secreted, apart from in the R-848 stimulated cells (**Figure 1B**). Conversely, TNF release which is independent of inflammasome activation, was unaffected (**Figure 1C**) (23).

**Figure 2:**
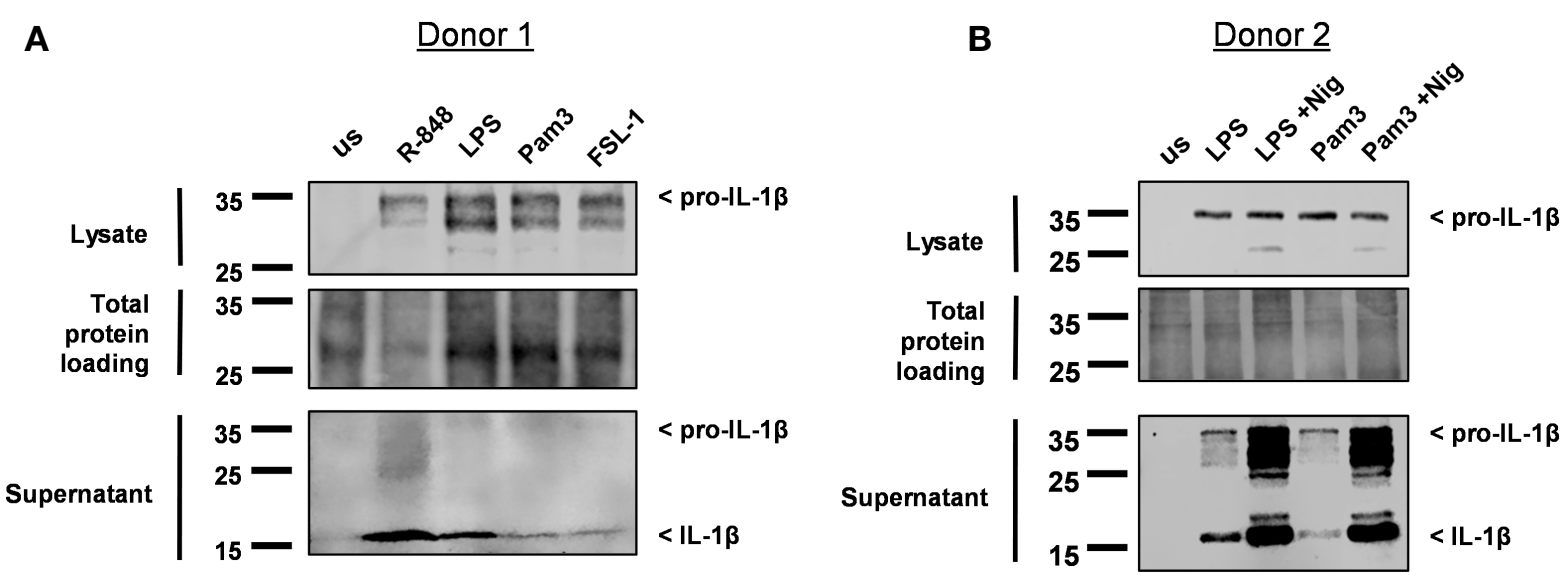
Monocytes release mature IL-1β into the cell supernatant following activation with Pam3, FSL-1, LPS and R-848. **(A)** Primary human monocytes were unstimulated (us) or stimulated for 24h with 100ng/ml Pam3, 1ng/ml FSL-1, 10ng/ml LPS or 2µg/ml R-848. **(B)** Monocytes were stimulated with LPS and Pam3 in the absence or the presence of 10µM nigericin (Nig) during the last 2h of stimulation. Western blot analysis for pro-IL-1β (31kDa) and mature IL-1β (17kDa) as well as total protein loading were performed. Blots are displayed from 2 individual donors.

Immunoblot analysis was then performed on cell lysates and supernatants to determine if the IL-1β released was pro-IL-1β or mature IL-1β. Detecting IL-1β in the supernatant was challenging, as these experiments were conducted in Optimem a reduced serum media, to reduce the background level of protein in the supernatant. Consequently, this led to a significant reduction in the already low level of IL-1β released from monocytes; particularly for the TLR2 ligands Pam3 and FSL-1 (**Supplementary Figure S1**). Pro-IL-1β was detected in the cell lysate following activation with all TLR ligands and could be detected in the supernatant for some of the donors (**Figure 2**, **Supplementary Figure S2**). Mature IL-1β was also visible in the supernatant of cells activated with Pam3, FSL-1, LPS and R-848. However, for Pam3 and FSL-1, mature IL-1β was not visible in the supernatant of all donors, probably due to the low level of mature IL-1β being at the limit of detection by western blotting (**Figure 2**, **Supplementary Figure S2**). In comparison, mature IL-1β was detected in the supernatants of all cells activated by TLR-ligands followed by nigericin for all donors (**Figure 2B**, **Supplementary Figure S2**). These results demonstrate that secretion of mature IL-1β from primary human monocytes following TLR activation alone, is not unique to TLR4.

### 3.2 IL-1β release is dependent on NLRP3 but does not require K^+^ efflux

**Figure 3:**
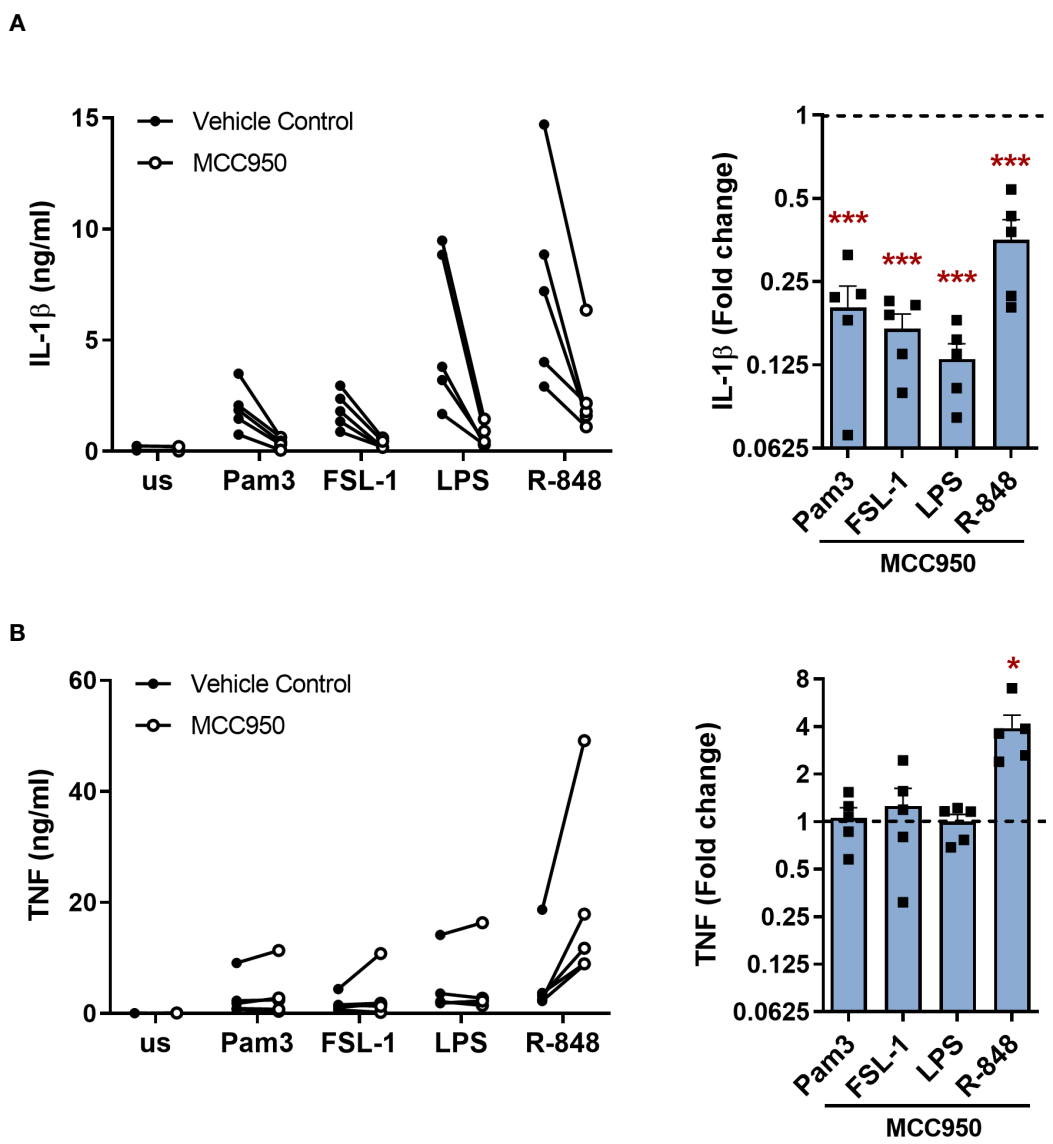
NLRP3 is required for TLR induced release of IL-1β in primary human monocytes. Monocytes were unstimulated (us) or stimulated for 24h with 100ng/ml Pam3, 1ng/ml FSL-1, 10ng/ml LPS or 2µg/ml R-848 in the presence of 10µM MCC950 or a vehicle control. Secretion of **(A)** IL-1β and **(B)** TNF were measured. Data are displayed as mean of technical triplicates (left) and as the fold change normalised to the corresponding TLR activation without MCC950 (dotted line) and pooled as the mean ± SEM (right) from 5 individual donors. Significance was determined using two-tailed one sample t-test against the response without MCC950 treatment (*p ≤ 0.05, ***p ≤ 0.001).

In the canonical NLRP3 inflammasome pathway, NLRP3 inflammasome assembly leads to activation of caspase-1 and consequent maturation of IL-1β, in addition to cleavage of GSDMD which promotes release of the mature IL-1β from the cell (4, 11, 13). To determine if NLRP3 activation was required for IL-1β release upon TLR stimulation alone, cells were treated with the selective NLRP3 inhibitor MCC950 (24). IL-1β secretion was significantly inhibited upon TLR activation in the presence of MCC950 compared to untreated cells (**Figure 3A**). TNF release was unaffected, except following stimulation with R-848 where TNF was significantly enhanced in the presence of MCC950 (**Figure 3B**). MCC950 alone did not affect the cell viability but was able to limit the reduction in viability of cells incubated with nigericin (**Supplementary Figures S3A, B**).

**Figure 4:**
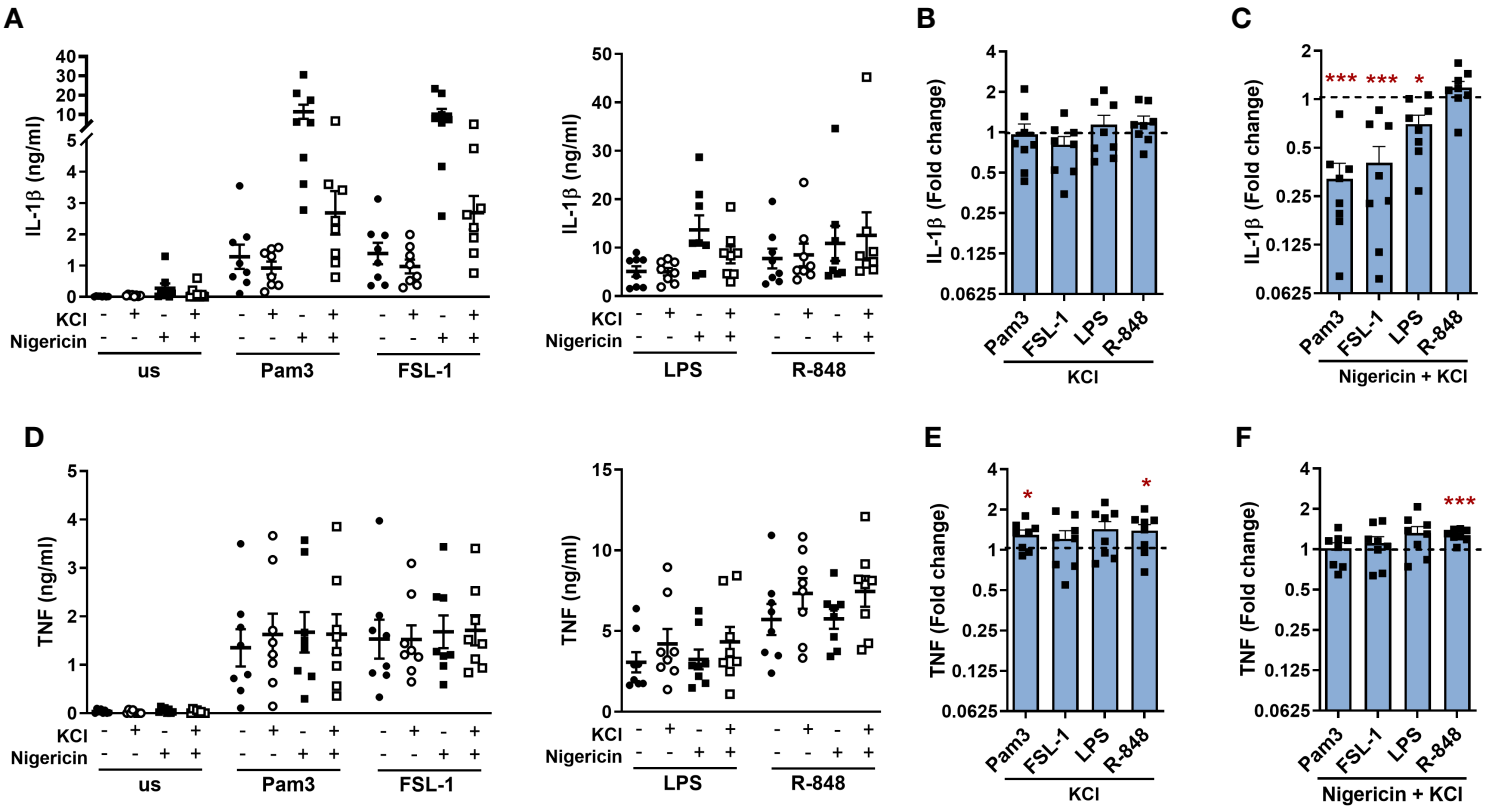
TLR induced IL-1β is not inhibited by extracellular KCl. Monocytes were unstimulated (us) or stimulated for 24h with 100ng/ml Pam3, 1ng/ml FSL-1, 10ng/ml LPS or 2µg/ml R-848 in the absence or presence of 20mM KCl. During the final 2h of stimulation, 10µM nigericin was added to the indicated wells. Secretion of **(A–C)** IL-1β and **(D–F)** TNF were measured. Data are displayed from 8 individual donors as the mean ± SEM **(A, D)** showing the level of cytokine secretion, **(B, E)** as the fold change normalised to the corresponding TLR activation in the absence of KCl (dotted line) and **(C, F)** as the fold change normalised to the corresponding TLR activation in the presence of nigericin without KCl (dotted line). Significance was determined using two-tailed one sample t-test against the response without nigericin treatment (*p ≤ 0.05, ***p ≤ 0.001).

As NLRP3 was activated by TLR stimulation in the absence of a second signal, further experiments were performed to explore if TLR induced IL-1β release required K^+^ or Cl^-^ efflux, which can activate the NLRP3 inflammasome (9, 25). Incubation in media containing KCl to impede K^+^ and Cl^-^ efflux, had no significant effect on the release of IL-1β (**Figures 4A, B**). However, in the presence of nigericin (a K^+^ ionophore), excess KCl significantly reduced the amplification of IL-1β secretion following activation of TLR1/2, 2/6 and 4 (**Figures 4A, C**). Following TLR stimulation alone, TNF secretion was moderately enhanced in the presence of excess KCl (**Figures 4D, E**). This was also evident in the presence of nigericin where R-848 induced TNF was significantly elevated (**Figure 4F**). Although incubation with nigericin reduced cell viability as would be expected due to induction of pyroptosis, KCl did not affect cell viability compared to the relevant controls (**Supplementary Figure S3C**). Together, these data demonstrate that IL-1β is released in a NLRP3 dependent manner that did not require K^+^ efflux.

### 3.3 TLR-induced IL-1β release is mediated by constitutively active caspase-1 and caspase-8

**Figure 5:**
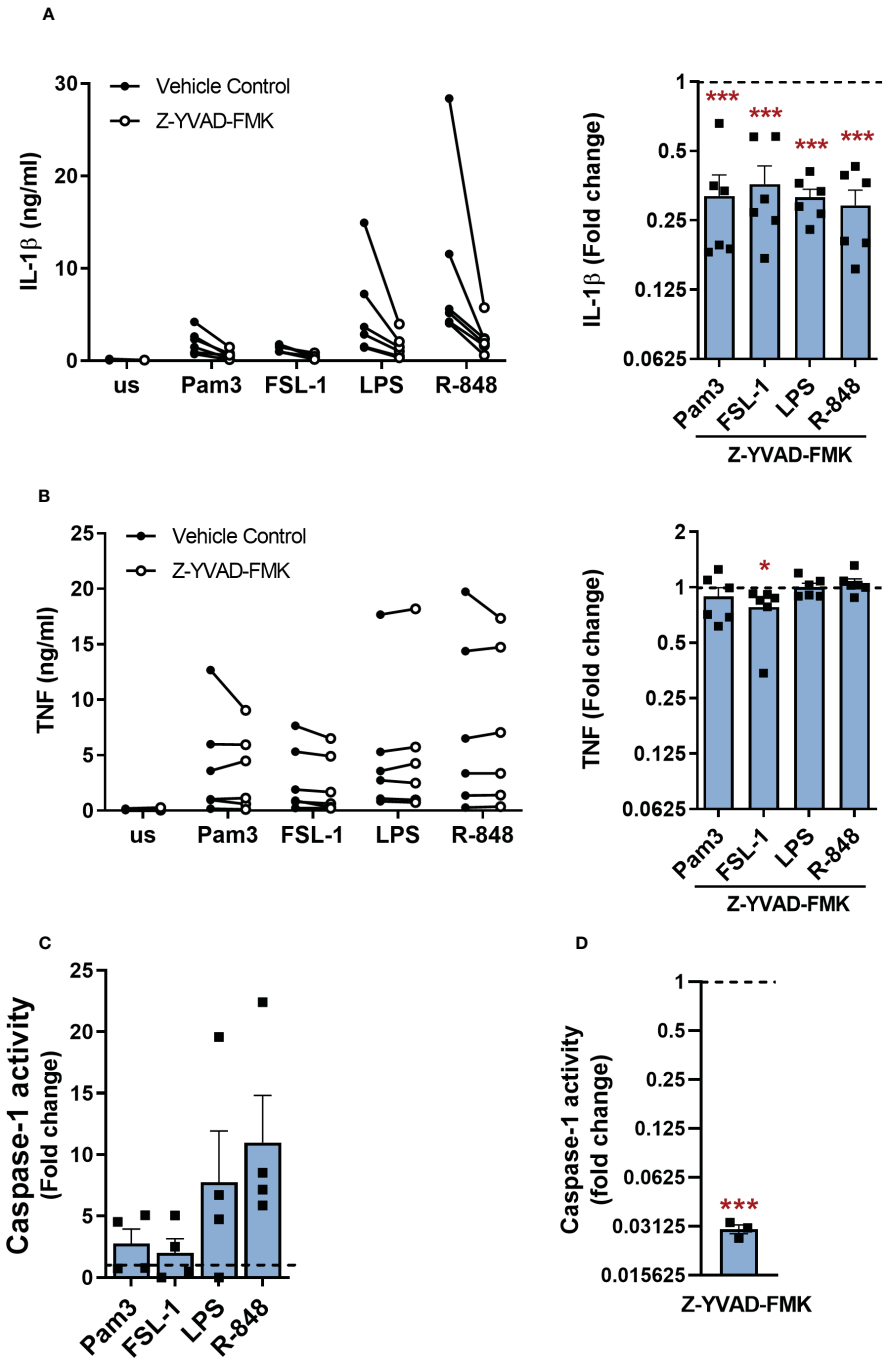
Caspase-1 is required for TLR-induced IL-1β release. **(A, B)** Primary human monocytes were unstimulated (us) or stimulated with 100ng/ml Pam3, 1ng/ml FSL-1, 10ng/ml LPS or 2µg/ml R-848 for 24h in the presence of 10µM Z-YVAD-FMK or a vehicle control. **(A)** IL-1β and **(B)** TNF secretion were measured. Data are displayed as mean of technical triplicates (left) and as the fold change normalised to the corresponding TLR activation without Z-YVAD-FMK (dotted line) as the mean ± SEM (right) from 6 individual donors. **(C)** Monocytes were unstimulated (us) or stimulated for 5.5h with 100ng/ml Pam3, 1ng/ml FSL-1, 10ng/ml LPS or 2µg/ml R-848 before measuring caspase-1 activity. Data are shown as fold change normalised to the unstimulated control (dotted line) as the mean ± SEM from 4 individual donors. **(D)** Monocytes were incubated with Z-YVAD-FMK for 4h before measuring caspase-1 activity. Data are shown as fold change normalised to cells in media alone (dotted line) and pooled as the mean ± SEM from 3 individual donors. Significance was determined using **(A, B)** a two-tailed one sample t-test or one sample Wilcoxon test against the response without inhibitor treatment or **(C)** Kruskal-Wallis test with posthoc Dunnett’s multiple comparisons test or **(D)** two-tailed one sample t-test against the response without inhibitor treatment (*p ≤ 0.05, ***p ≤ 0.001).

NLRP3 inflammasome activation results in cleavage and activation of caspase-1, which in turn cleaves pro-IL-1β to its mature form and GSDMD leading to pore formation in the cell membrane (4, 11, 13). Accordingly, when monocytes were stimulated with TLR ligands in the presence of the caspase-1 inhibitor Z-YVAD-FMK, IL-1β secretion was significantly suppressed (**Figure 5A**). TNF was mostly unaltered in all apart from FSL-1 activated cells where a modest but significant reduction was observed (**Figure 5B**). Incubation with Z-YVAD-FMK did not affect the cell viability (**Supplementary Figure S3D**).

These results indicated that caspase-1 was active even in the absence of a NLRP3-activating second signal, thus caspase-1 activity was measured to determine if TLR activation alone led to increased activity. Although a trend towards enhanced caspase-1 activity was observed following stimulation with all TLRs tested compared to unstimulated cells, these levels did not reach significance (**Figure 5C**). Caspase-1 has previously been reported to be constitutively active in primary human monocytes, thus unstimulated cells were next treated with the caspase-1 inhibitor Z-YVAD-FMK to confirm if there was a constitutive level of activation (26). Inhibition of caspase-1 in resting unstimulated cells led to a significant decrease in caspase-1 activity (**Figure 5D**).

**Figure 6:**
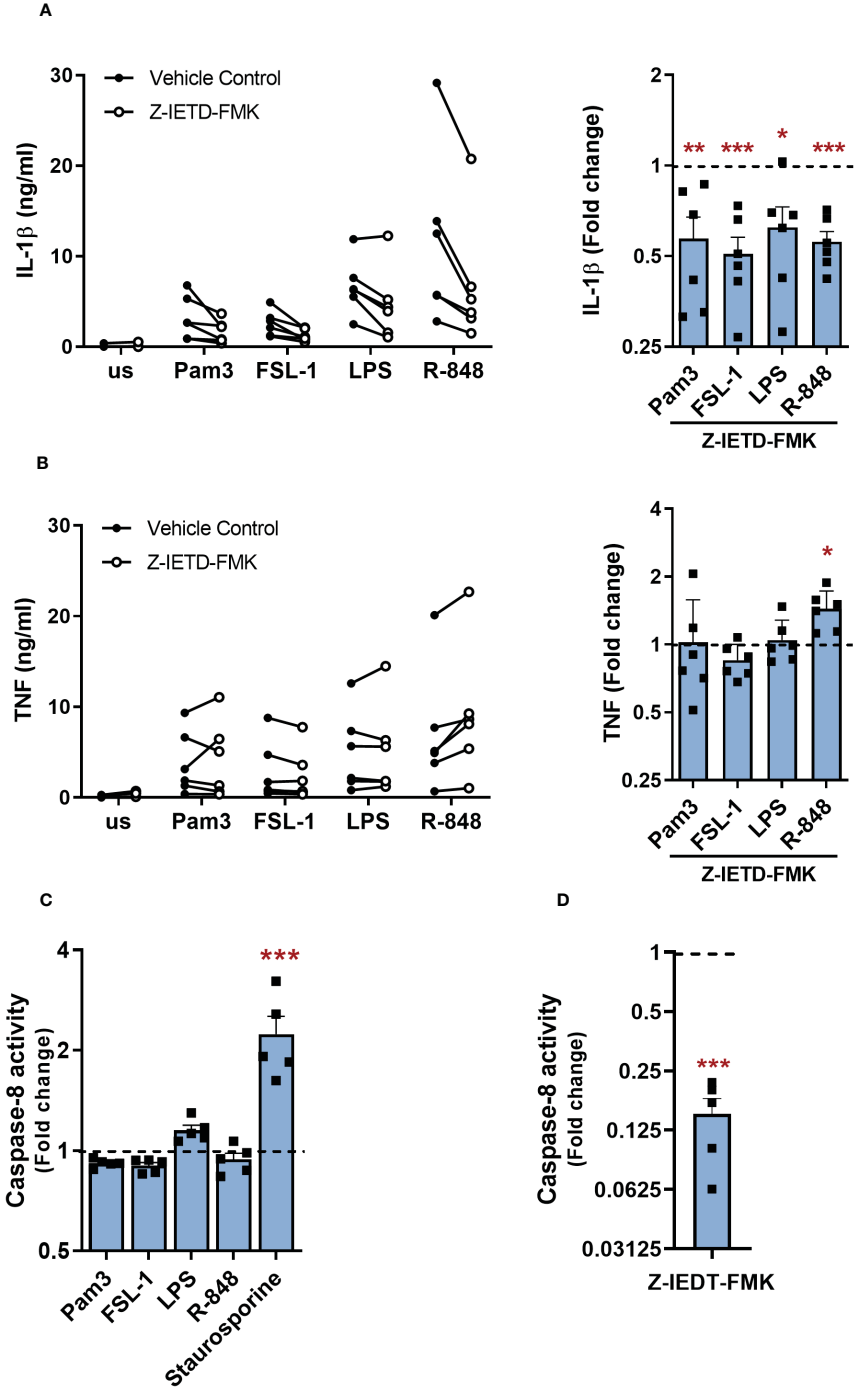
Constitutively active caspase-8 is required for TLR-induced IL-1β release. **(A, B)** Primary human monocytes were unstimulated (us) or stimulated with 100ng/ml Pam3, 1ng/ml FSL-1, 10ng/ml LPS or 2µg/ml R-848 in the presence of 1µM Z-IETD-FMK or a vehicle control for 24h. **(A)** IL-1β and **(B)** TNF secretion were measured by ELISA. Data are displayed as mean of technical triplicates (left) and as the fold change normalised to the corresponding TLR activation without Z-IETD-FMK (dotted line) and pooled as the mean ± SEM (right) from 6 individual donors. **(C)** Cells were unstimulated (us) or stimulated with 100ng/ml Pam3, 1ng/ml FSL-1, 10ng/ml LPS, 2µg/ml R-848 or 1µM Staurosporine for 6h to measure caspase-8 activity. Data are shown as fold change normalised to the unstimulated control (dotted line) as the mean ± SEM from 5 individual donors. **(D)** Unstimulated monocytes were incubated with Z-IETD-FMK for 6h before measuring caspase-8 activity. Data are shown as fold change normalised to cells in vehicle control (dotted line) and pooled as the mean ± SEM from 5 individual donors. Significance was determined using **(A, B)** two-tailed one sample t-test against the response without inhibitor treatment or **(C)** one-way ANOVA using with posthoc Dunnett’s multiple comparisons test or **(D)** two-tailed one sample t-test against the response without inhibitor treatment (*p ≤ 0.05, **p ≤ 0.01, ***p ≤ 0.001).

In the alternative NLRP3 inflammasome pathway, caspase-8 was required for TLR4 induced NLRP3 activation (19). To investigate if caspase-8 is also required by other TLRs for the secretion of IL-1β from primary human monocytes, cells were treated with 1µM of the caspase-8 inhibitor Z-IETD-FMK 30 min before stimulation with TLR ligands. Catalytic inhibition of caspase-8 significantly reduced IL-1β release for all TLRs (**Figure 6A**). However, TNF secretion was unaffected in all except R848-activated cells where the caspase-8 inhibitor led on average to a 1.4 fold higher secretion of TNF (**Figure 6B**). Cell viability was not compromised upon caspase-8 inhibitor treatment (**Supplementary Figure S3E**). A caspase-8 activity assay was then performed to assess whether TLR stimulation triggers the activation of caspase-8. Staurosporine, a known activator of caspase-8, induced a significant increase in caspase-8 activity but, interestingly, TLR stimulation did not enhance caspase-8 activity (**Figure 6C**) (27). To evaluate whether there was a constitutive level of caspase-8 activity in monocytes, similar to that observed for caspase-1, unstimulated cells were treated with the caspase-8 inhibitor Z-IETD-FMK, which led to a significant decrease in caspase-8 activity (**Figure 6D**). These findings suggest that constitutive caspase-1 and caspase-8 activity are sufficient to facilitate IL-1β release following TLR activation of human monocytes.

### 3.4 TLRs activate caspase-1 and IL-1β secretion independently of RIPK1 kinase activity

**Figure 7:**
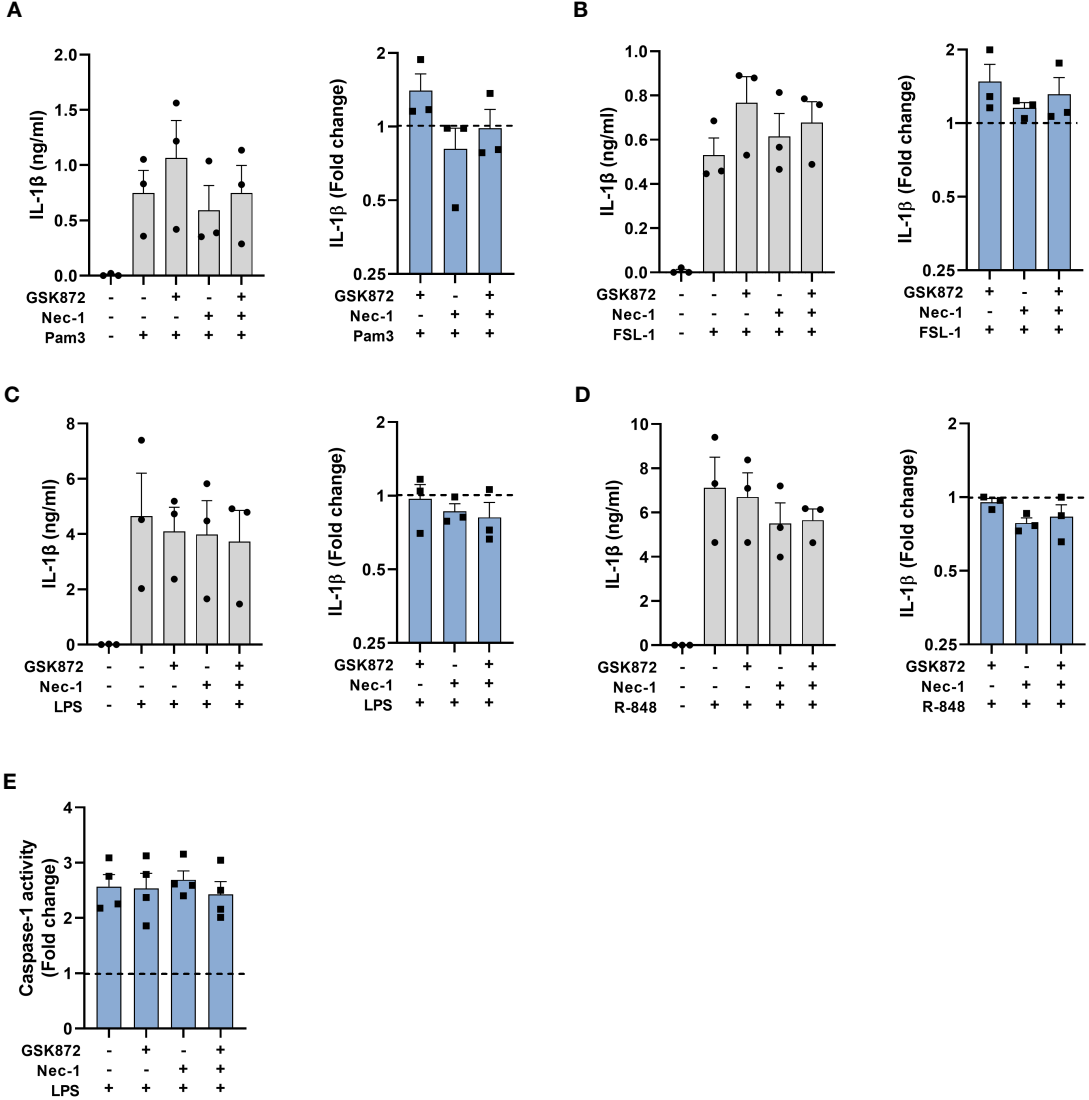
RIPK1 kinase activity is not required for caspase-1 activation and IL-1β secretion following TLR stimulation. Primary human monocytes were unstimulated or stimulated for **(A–D)** 20h or **(E)** 5.5h, with **(A)** 100ng/ml Pam3, **(B)** 1ng/ml FSL-1, **(C, E)** 10ng/ml LPS or **(D)** 2µg/ml R-848 in the presence of 40µM necrostatin-1 (Nec-1) or 2.5 µM GSK872. **(A–D)** Data are displayed from 3 individual donors as the mean ± SEM, for IL-1β secretion and as the fold change normalised to the IL-1β release from TLR activation alone (dotted line). **(E)** Caspase-1 activity is displayed as the fold change normalised to LPS activation alone (dotted line) as the mean ± SEM from 4 individual donors.

In addition to caspase-8, RIPK1 and FADD are also suggested to be required upstream of NLRP3 activation within the alternative pathway in BLaER1 cells. However, in BLaER1 cells the dependence on RIPK1 could only be observed in necroptosis deficient cells, as disruption of RIPK1 led to a necroptotic release of IL-1β (19). To investigate the requirement for RIPK1 in primary human monocytes, cells were therefore stimulated with TLR ligands in the presence of the RIPK1 inhibitor Nec-1 with or without GSK872, an inhibitor of RIPK3 to prevent necroptosis (**Figure 7**). Inhibition of RIPK1 kinase activity with Nec-1 alone did not affect IL-1β release and did not induce LDH release (**Figures 7A–D**, **Supplementary Figure S4**). In addition, IL-1β secretion was not significantly affected in the presence of dual inhibition with Nec-1 and GSK872 (**Figures 7A–D**). Furthermore, no effect was observed on LPS induced caspase-1 activation by these inhibitors when used alone or in combination (**Figure 7E**).

### 3.5 TLR-induced IL-1β is released independently of pyroptosis

**Figure 8:**
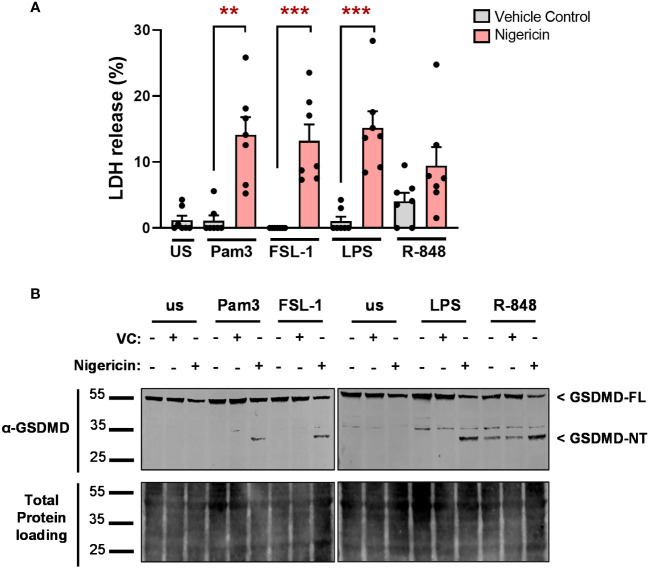
TLR-induced IL-1β release is independent of pyroptosis. Primary human monocytes were unstimulated (us) or stimulated for 24h with 100ng/ml Pam3, 1ng/ml FSL-1, 10ng/ml LPS or 2µg/ml R-848 in the presence of 10µM nigericin or a vehicle control (VC). **(A)** Pyroptosis was measured with a lactate dehydrogenase (LDH) release assay. Data are displayed as mean ± SEM of pooled data from 7 individual donors. Significance was determined using Friedman test (**p ≤ 0.01, ***p ≤ 0.001). **(B)** Gasdermin D (GSDMD-FL, 53kDa), gasdermin N-terminus (GSDMD-NT, 31kDa) and total protein loading were assessed by western blot. Western blots are representative of 3 individual donors.

In the canonical NLRP3 inflammasome pathway, the release of IL-1β is facilitated by the formation of GSDMD pores, which also induce pyroptosis (11, 13, 14). However, in the alternative NLRP3 pathway, TLR4-induced IL-1β is independent of pyroptosis (19). Thus, the involvement of pyroptotic cell death was assessed by measuring LDH release upon TLR stimulation. Pam3, FSL-1, LPS or R-848 alone, did not significantly increase LDH release compared to unstimulated monocytes. Although stimulation of cells with R-848 moderately increased LDH levels in some donors, this did not reach statistical significance. When incubated with nigericin, LDH release increased for all conditions (**Figure 8A**). This corresponded with cleavage of 53kDa full length GSDMD (GSDMD-FL) to the 31kDa pore-forming N-terminus (GSDMD-NT) for all cells stimulated with TLR ligands in the presence of nigericin. In cells stimulated in the absence of nigericin, only R-848 led to detectable GSDMD cleavage (**Figure 8B**, **Supplementary Figure S5**). This suggests that TLR induced IL-1β secretion is independent of pyroptosis.

### 3.6 Inhibition of GSDMD pore formation reduced TLR-induced IL-1β release

**Figure 9:**
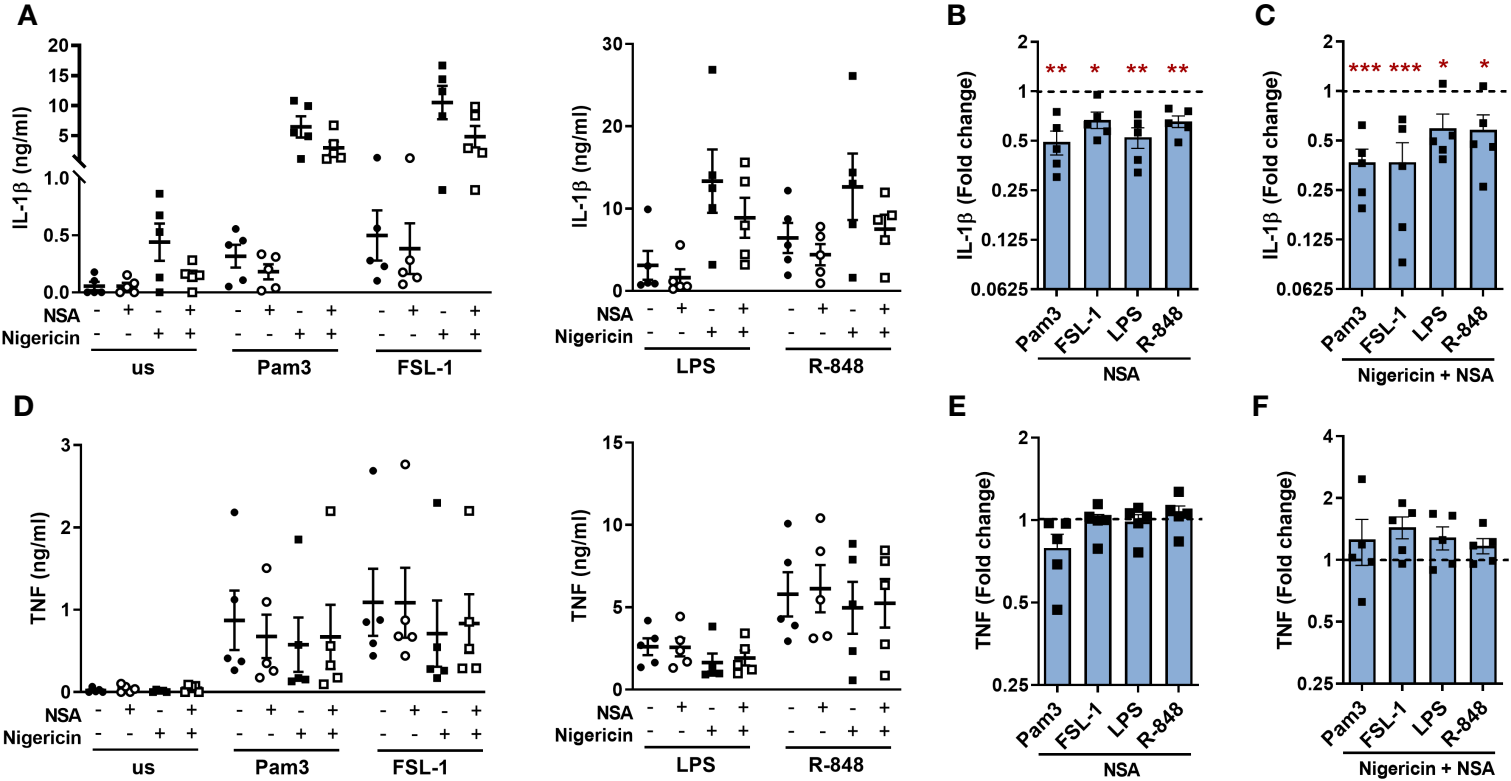
Necrosulfonamide inhibits TLR induced IL-1β released via the alternative pathway. Primary human monocytes were unstimulated (us) or stimulated for 5.5h with 100ng/ml Pam3, 1ng/ml FSL-1, 10ng/ml LPS or 2µg/ml R-848 in the absence or presence of 10µM necrosulfonamide (NSA) and/or 10µM nigericin for the last 2h of stimulation. Secretion of **(A)** IL-1β and **(B)** TNF were measured. Data are displayed from 5 individual donors as the mean ± SEM **(A, D)** showing the level of cytokine secretion, **(B, E)** the fold change normalised to the corresponding TLR activation without NSA (dotted line) and **(C, F)** the fold change normalised to the corresponding TLR activation with nigericin but without NSA (dotted line). **(B, C, E, F)** Significance was determined using two-tailed one sample t-test against the response without NSA treatment (*p ≤ 0.05, **p ≤ 0.01), ***p ≤ 0.001.

As R-848, the most potent TLR ligand used in this study, induced GSDMD cleavage, it was possible that the other ligands may also cleave GSDMD but to a lower level than detected by immunoblotting. Thus, an alternative approach was explored using necrosulfonamide (NSA), which directly binds to GSDMD and inhibits the p30-GSDMD oligomerization in the membrane (28). NSA significantly suppressed IL-1β release for all TLRs tested in both the presence or absence of nigericin (**Figures 9A–C**). Conversely, secretion of TNF from TLR stimulated monocytes was not significantly affected but did show a small increase in cells incubated with nigericin (**Figures 9D–F**). To ensure that these results were not due to an off-target effect of NSA on the induction of pro-IL-1β or NLRP3 upregulation, the gene expression of *IL1B* and *NLRP3* were measured following TLR1/2 and TLR4 activation. Both genes were upregulated following TLR stimulation, but this was not affected by NSA (**Supplementary Figure S6**). Cell viability of TLR activated monocytes was also unaffected by NSA and the reduction in cell viability in cells incubated with nigericin was partially reversed in the presence of NSA (**Supplementary Figure S3F**). These data suggest that the release of TLR-induced IL-1β may in part be facilitated by GSDMD pores.

## 4 Discussion

Primary human monocytes activate an alternative NLRP3 inflammasome pathway upon stimulation of TLR4. Unlike the canonical pathway, the alternative pathway does not require a secondary activation signal. Furthermore, using BLaER1 cells (immortalised B lymphocytes transdifferentiated to a monocytic like phenotype) as a model for human monocytes, TRIF, RIPK1, FADD and CASP8 were identified as signalling molecules upstream of NLRP3 in the alternative pathway (19). Intriguingly, several studies have previously demonstrated that TLR2, TLR7 and TLR8 ligands can also induce secretion of IL-1β from primary human monocytes without the need for a secondary NLRP3 activating signal (26, 29–34). However, as only TLR3 and TLR4 are known to engage the adaptor protein TRIF in their signalling cascades, this study set out to investigate if other TLRs could activate the alternative pathway of IL-1β secretion in primary human monocytes (20).

Activation of TLR1/2, TLR2/6, TLR4 and TLR7 and/or TLR8 (with the dual TLR7/TLR8 ligand R-848) induced NLRP3 dependent secretion of IL-1β in the absence of a secondary signal, suggesting that the alternative NLRP3 pathway is a common feature of TLR signalling in primary human monocytes. Addition of nigericin to introduce a secondary signal (as used by the canonical NLRP3 pathway), amplified IL-1β secretion following activation with all TLR ligands apart from R-848. It appeared that R-848 alone was sufficient to induce the release of a maximal level of IL-1β. Interestingly, R-848 has recently been suggested to induce formation of a heterodimer with TLR4 in overexpression studies in HEK293 cells, possibly leading to an amplification of downstream signalling (35). However, it is not yet known if this occurs in primary human monocytes.

As IL-1β lacks a signal sequence, it is not secreted via the endoplasmic reticulum-Golgi route (12). In the canonical NLRP3 pathway, GSDMD pores that form following cleavage of GSDMD by caspase-1, facilitate IL-1β release and initiate pyroptosis (13, 36). However, as pyroptosis is not associated with TLR4 stimulation, release of IL-1β in the alternative pathway is believed to be via an unconventional secretion pathway (37). Several studies have reported alternative secretion mechanisms for IL-1β in which GSDMD and pyroptosis are not required. In human monocytes, TLR4 induced IL-1β secretion has been observed from LAMP2A^+^ vesicles in a process independent of pyroptosis and at a slower rate than occurs through GSDMD pores (38). In macrophages where the canonical pathway is in operation, knockdown of early endosome autoantigen 1 has been observed to decrease IL-1β release without affecting pyroptosis (39).

In our study, only activation with R-848 led to detectable cleavage of GSDMD. However, incubation with NSA (an inhibitor of GSDMD oligomerisation) reduced IL-1β release induced by all TLR ligands without affecting upregulation of *IL1B* or *NLRP3*, indicating that GSDMD pores may contribute to IL-1β release, but at a level below detection by immunoblotting. Furthermore, none of the TLRs activated led to a significant increase in LDH release, suggesting TLR induced IL-1β secretion is independent of pyroptosis (19). It may be that low level GSDMD pore formation is not sufficient to initiate pyroptosis in monocytes due to membrane repair mechanisms. ESCRTIII-dependent membrane repair has been reported to delay or prevent GSDMD mediated cell death in several cell types (40). As expected, addition of nigericin did increase LDH release and GSDMD cleavage following activation of all TLRs, which was associated with an increase in IL-1β secretion. However, GSDMD cleavage above that induced by R-848 alone, appeared to be beyond the level required for full IL-1β release.

Similar to the initial studies of TLR4 activation of the alternative pathway, IL-1β secretion was dependent on caspase-1 activity but independent of K^+^ efflux for all TLRs. Caspase-1 was found to be constitutively active, as has previously been shown by Netea et al., in primary human monocytes (26). In our study, TLR activation exhibited a trend towards increased caspase-1 activity, but this did not reach significance compared to unstimulated cells, possibly due to the variation in donor responses. However, chemical inhibition of caspase-1 led to a substantial decrease in IL-1β secretion.

Both K^+^ and Cl^-^ efflux have been demonstrated to activate the NLRP3 inflammasome (9, 25). However, stimulation of monocytes in media containing increased KCl had no effect on TLR induced IL-1β but did decrease the additional IL-1β released in the presence of nigericin. Activation of primary human monocytes with nigericin has previously been shown to be sufficient to induce canonical inflammasome assembly dependent on K^+^ and Cl^-^ efflux in the absence of priming (41). Furthermore, as K^+^ efflux was not required for LPS induced IL-1β secretion, this also suggested that LPS had not entered the cytoplasm to activate the non-canonical pathway.

Caspase-8 is suggested to provide an alternative route to inflammasome activation, but how it activates NLRP3 has not yet been defined but is speculated to involve an unknown protein downstream of caspase-8 (19, 37). Intriguingly, TLR activation did not increase caspase-8 activity compared to unstimulated cells in our study, which is in contrast to the report of increased caspase-8 cleavage upon LPS stimulation of primary human monocytes and BLaER1 cells (19). It appeared that primary human monocytes exhibit a constitutive level of caspase-8 activity, which when inhibited led to a partial but significant inhibition of IL-1β secretion. However, the caspase-8 inhibitor Z-IETD-FMK has been reported to partially inhibit caspase-1 as an off-target effect, making it difficult to determine if the effect of Z-IETD-FMK on IL-1β release was due to a direct effect on caspase-8 (42). Thus, a different approach was taken to examine the mechanism of NLRP3 activation. RIPK1 is also proposed to act upstream of NLRP3 in the alternative pathway in BLaER1 cells. However, in our study inhibition of RIPK1 kinase activity did not affect TLR induced caspase-1 activation or IL-1β secretion in primary human monocytes. However, this does not preclude a potential role for RIPK1 that is distinct from its kinase activity.

On the whole, our study agrees with the primary monocyte data presented in the original study that discovered TLR4 activation of the alternative NLRP3 pathway (19). Our results confirm that TLR4 activates IL-1β processing and release from human monocytes in a K^+^ independent but caspase-1 dependent manner. However, our data demonstrate that this pathway is common to other TLRs, not just TLR4 and is not dependent on RIPK1 kinase activity in primary human monocytes. Although the NLRP3, caspase-1 and caspase-8 inhibition data measured IL-1β release by ELISA, which detects secretion of both pro-IL-1β and mature IL-1β, secretion of mature IL-1β was confirmed following activation of TLR1/2, TLR2/6 and TLR7 and/or 8 by western blotting. This then calls into question whether TRIF is a necessary component of the alternative pathway or simply a contributing, but non-essential factor downstream of those TLRs that use TRIF as an adaptor protein. To fully investigate the signalling events upstream of NLRP3 activation in primary human monocytes is not a trivial undertaking, as monocytes do not remain viable in culture long enough to permit knockdown experiments. Attempting to overcome this by differentiating the cells to macrophages would not provide a solution, as the cells consequently lose the ability to activate the alternative pathway; human macrophages require canonical NLRP3 activation to induce IL-1β secretion. This explains why studies exploring the alternative pathway have relied on chemical inhibitors or the use of BLaER1 cells as a model for human monocytes. Nevertheless, our data expand upon the current knowledge of the alternative NLRP3 pathway, demonstrating that this pathway is common to multiple TLRs, has the potential to release IL-1β via GSDMD pores independently of pyroptosis alongside other unconventional secretory pathways and suggest that the pathway upstream of NLRP3 activation can function independently of TRIF and RIPK1 kinase activity in primary human monocytes.

## Data availability statement

The original contributions presented in the study are included in the article/**Supplementary Material**. Further inquiries can be directed to the corresponding author.

## Ethics statement

The studies involving humans were approved by Wales Research Ethics Committee 6. The studies were conducted in accordance with the local legislation and institutional requirements. The participants provided their written informed consent to participate in this study.

## Author contributions

SU, LM and SS were responsible for acquisition of data and data analysis. SU, LM, MF, and SS were responsible for study design and interpretation. SS developed the study concept, obtained funding and ethics. All authors were involved in drafting the manuscript or revising it critically for important intellectual content. All authors contributed to the article and approved the submitted version.

## References

[1] Dinarello CA. A clinical perspective of IL-1β as the gatekeeper of inflammation. Eur J Immunol (2011) 41(5):1203–17. doi: 10.1002/eji.201141550 21523780

[2] Sahoo M Ceballos-Olvera I del Barrio L Re F. Role of the inflammasome, IL-1β, and IL-18 in bacterial infections. ScientificWorldJournal (2011) 11:2037–50. doi: 10.1100/2011/212680 PMC3217589 22125454

[3] Unterberger S Davies KA Rambhatla SB Sacre S. Contribution of toll-like receptors and the NLRP3 inflammasome in rheumatoid arthritis pathophysiology. Immunotargets Ther (2021) 10:285–98. doi: 10.2147/ITT.S288547 PMC8326786 34350135

[4] Martinon F Burns K Tschopp J. The inflammasome: A molecular platform triggering activation of inflammatory caspases and processing of proIL-β. Mol Cell (2002) 10(2):417–26. doi: 10.1016/S1097-2765(02)00599-3 12191486

[5] Sharma D Kanneganti TD. The cell biology of inflammasomes: Mechanisms of inflammasome activation and regulation. J Cell Biol (2016) 213(6):617–29. doi: 10.1083/jcb.201602089 PMC4915194 27325789

[6] He Y Hara H Núñez G. Mechanism and regulation of NLRP3 inflammasome activation. Trends Biochem Sci (2016) 41(12):1012–21. doi: 10.1016/j.tibs.2016.09.002 PMC5123939 27669650

[7] Bauernfeind FG Horvath G Stutz A Alnemri ES MacDonald K Speert D. Cutting edge: NF-kappaB activating pattern recognition and cytokine receptors license NLRP3 inflammasome activation by regulating NLRP3 expression. J Immunol (Baltimore Md 1950) (2009) 183(2):787–91. doi: 10.4049/jimmunol.0901363 PMC2824855 19570822

[8] Lu A Wu H. Structural mechanisms of inflammasome assembly. FEBS J (2015) 282(3):435–44. doi: 10.1111/febs.13133 PMC6400279 25354325

[9] Muñoz-Planillo R Kuffa P Martínez-Colón G Smith BL Rajendiran TM Núñez G. K(+) efflux is the Common Trigger of NLRP3 inflammasome Activation by Bacterial Toxins and Particulate Matter. Immunity (2013) 38(6):1142–53. doi: 10.1016/j.immuni.2013.05.016 PMC3730833 23809161

[10] Franchi L Eigenbrod T Núñez G. Cutting edge: TNF-alpha mediates sensitization to ATP and silica via the NLRP3 inflammasome in the absence of microbial stimulation. J Immunol (Baltimore Md 1950) (2009) 183(2):792–6. doi: 10.4049/jimmunol.0900173 PMC2754237 19542372

[11] He W-t Wan H Hu L Chen P Wang X Huang Z. Gasdermin D is an executor of pyroptosis and required for interleukin-1β secretion. Cell Res (2015) 25(12):1285–98. doi: 10.1038/cr.2015.139 PMC4670995 26611636

[12] Rubartelli A Cozzolino F Talio M Sitia R. A novel secretory pathway for interleukin-1 beta, a protein lacking a signal sequence. EMBO J (1990) 9(5):1503–10. doi: 10.1002/j.1460-2075.1990.tb08268.x PMC551842 2328723

[13] Evavold CL Ruan J Tan Y Xia S Wu H Kagan JC. The pore-forming protein gasdermin D regulates interleukin-1 secretion from living macrophages. Immunity (2018) 48(1):35–44.e6. doi: 10.1016/j.immuni.2017.11.013 29195811 PMC5773350

[14] Shi J Zhao Y Wang K Shi X Wang Y Huang H. Cleavage of GSDMD by inflammatory caspases determines pyroptotic cell death. Nature (2015) 526:660. doi: 10.1038/nature15514 26375003

[15] Kayagaki N Stowe IB Lee BL O’Rourke K Anderson K Warming S. Caspase-11 cleaves gasdermin D for non-canonical inflammasome signalling. Nature (2015) 526(7575):666–71. doi: 10.1038/nature15541 26375259

[16] Shi J Zhao Y Wang Y Gao W Ding J Li P. Inflammatory caspases are innate immune receptors for intracellular LPS. Nature (2014) 514(7521):187–92. doi: 10.1038/nature13683 25119034

[17] Baker PJ Boucher D Bierschenk D Tebartz C Whitney PG D'Silva DB. NLRP3 inflammasome activation downstream of cytoplasmic LPS recognition by both caspase-4 and caspase-5. Eur J Immunol (2015) 45(10):2918–26. doi: 10.1002/eji.201545655 26173988

[18] Rühl S Broz P. Caspase-11 activates a canonical NLRP3 inflammasome by promoting K+ efflux. Eur J Immunol (2015) 45(10):2927–36. doi: 10.1002/eji.201545772 26173909

[19] Gaidt Moritz M Ebert Thomas S Chauhan D Schmidt T Schmid-Burgk Jonathan L Rapino F. Human monocytes engage an alternative inflammasome pathway. Immunity (2016) 44(4):833–46. doi: 10.1016/j.immuni.2016.01.012 27037191

[20] Jenkins KA Mansell A. TIR-containing adaptors in Toll-like receptor signalling. Cytokine (2010) 49(3):237–44. doi: 10.1016/j.cyto.2009.01.009 19264502

[21] Bøyum A. Separation of blood leucocytes, granulocytes and lymphocytes. Tissue Antigens (1974) 4(3):269–74. 4415728

[22] Menck K Behme D Pantke M Reiling N Binder C Pukrop T. Isolation of human monocytes by double gradient centrifugation and their differentiation to macrophages in teflon-coated cell culture bags. J Vis Exp (2014) 91):e51554-e. doi: 10.3791/51554-v PMC4828059 25226391

[23] Lieu ZZ Lock JG Hammond LA La Gruta NL Stow JL Gleeson PA. A trans-Golgi network golgin is required for the regulated secretion of TNF in activated macrophages *in vivo* . Proc Natl Acad Sci (2008) 105(9):3351–6. doi: 10.1073/pnas.0800137105 PMC2265200 18308930

[24] Coll RC Robertson AAB Chae JJ Higgins SC Muñoz-Planillo R Inserra MC. A small-molecule inhibitor of the NLRP3 inflammasome for the treatment of inflammatory diseases. Nat Med (2015) 21(3):248–55. doi: 10.1038/nm.3806 PMC4392179 25686105

[25] Tang T Lang X Xu C Wang X Gong T Yang Y. CLICs-dependent chloride efflux is an essential and proximal upstream event for NLRP3 inflammasome activation. Nat Commun (2017) 8(1):202. doi: 10.1038/s41467-017-00227-x 28779175 PMC5544706

[26] Netea MG Nold-Petry CA Nold MF Joosten LAB Opitz B van der Meer JHM. Differential requirement for the activation of the inflammasome for processing and release of IL-1beta in monocytes and macrophages. Blood (2009) 113(10):2324–35. doi: 10.1182/blood-2008-03-146720 PMC2652374 19104081

[27] Stepczynska A Lauber K Engels IH Janssen O Kabelitz D Wesselborg S. Staurosporine and conventional anticancer drugs induce overlapping, yet distinct pathways of apoptosis and caspase activation. Oncogene (2001) 20(10):1193–202. doi: 10.1038/sj.onc.1204221 11313863

[28] Rathkey JK Zhao J Liu Z Chen Y Yang J Kondolf HC. Chemical disruption of the pyroptotic pore-forming protein gasdermin D inhibits inflammatory cell death and sepsis. Sci Immunol (2018) 3(26):eaat2738. doi: 10.1126/sciimmunol.aat2738 30143556 PMC6462819

[29] Thwaites RS Unterberger S Chamberlain G Gray H Jordan K Davies KA. Expression of sterile-α and armadillo motif containing protein (SARM) in rheumatoid arthritis monocytes correlates with TLR2-induced IL-1β and disease activity. Rheumatol (Oxford) (2021) 60(12):5843–53. doi: 10.1093/rheumatology/keab162 PMC8645275 33605409

[30] Alberts BM Barber JS Sacre SM Davies KA Ghezzi P Mullen LM. Precipitation of soluble uric acid is necessary for *in vitro* activation of the NLRP3 inflammasome in primary human monocytes. J Rheumatol (2019) 46(9):1141–50. doi: 10.3899/jrheum.180855 30824640

[31] Snodgrass RG Huang S Choi IW Rutledge JC Hwang DH. Inflammasome-mediated secretion of IL-1β in human monocytes through TLR2 activation; modulation by dietary fatty acids. J Immunol (2013) 191(8):4337–47. doi: 10.4049/jimmunol.1300298 PMC3825708 24043885

[32] Funderburg NT Jadlowsky JK Lederman MM Feng Z Weinberg A Sieg SF. The Toll-like receptor 1/2 agonists Pam(3) CSK(4) and human β-defensin-3 differentially induce interleukin-10 and nuclear factor-κB signalling patterns in human monocytes. Immunology (2011) 134(2):151–60. doi: 10.1111/j.1365-2567.2011.03475.x PMC3194223 21896010

[33] Hurst J Prinz N Lorenz M Bauer S Chapman J Lackner KJ. TLR7 and TLR8 ligands and antiphospholipid antibodies show synergistic effects on the induction of IL-1beta and caspase-1 in monocytes and dendritic cells. Immunobiology (2009) 214(8):683–91. doi: 10.1016/j.imbio.2008.12.003 19249118

[34] Cros J Cagnard N Woollard K Patey N Zhang S-Y Senechal B. Human CD14dim monocytes patrol and sense nucleic acids and viruses via TLR7 and TLR8 receptors. Immunity (2010) 33(3):375–86. doi: 10.1016/j.immuni.2010.08.012 PMC3063338 20832340

[35] Thada S Horvath GL Müller MM Dittrich N Conrad ML Sur S. Interaction of TLR4 and TLR8 in the innate immune response against mycobacterium tuberculosis. Int J Mol Sci (2021) 22(4):1560. doi: 10.3390/ijms22041560 33557133 PMC7913854

[36] Sborgi L Rühl S Mulvihill E Pipercevic J Heilig R Stahlberg H. GSDMD membrane pore formation constitutes the mechanism of pyroptotic cell death. EMBO J (2016) 35(16):1766–78. doi: 10.15252/embj.201694696 PMC5010048 27418190

[37] Gaidt MM Hornung V. Alternative inflammasome activation enables IL-1β release from living cells. Curr Opin Immunol (2017) 44:7–13. doi: 10.1016/j.coi.2016.10.007 27842238 PMC5894802

[38] Semino C Carta S Gattorno M Sitia R Rubartelli A. Progressive waves of IL-1β release by primary human monocytes via sequential activation of vesicular and gasdermin D-mediated secretory pathways. Cell Death Dis (2018) 9(11):1088. doi: 10.1038/s41419-018-1121-9 30352992 PMC6199333

[39] Baroja-Mazo A Compan V Martín-Sánchez F Tapia-Abellán A Couillin I Pelegrín P. Early endosome autoantigen 1 regulates IL-1β release upon caspase-1 activation independently of gasdermin D membrane permeabilization. Sci Rep (2019) 9(1):5788. doi: 10.1038/s41598-019-42298-4 30962463 PMC6453936

[40] Rühl S Shkarina K Demarco B Heilig R Santos JC Broz P. ESCRT-dependent membrane repair negatively regulates pyroptosis downstream of GSDMD activation. Science (2018) 362(6417):956–60. doi: 10.1126/science.aar7607 30467171

[41] Gritsenko A Yu S Martin-Sanchez F Diaz-del-Olmo I Nichols E-M Davis DM. Priming is dispensable for NLRP3 inflammasome activation in human monocytes *in vitro* . Front Immunol (2020) 11. doi: 10.3389/fimmu.2020.565924 PMC7555430 33101286

[42] Buscetta M Di Vincenzo S Miele M Badami E Pace E Cipollina C. Cigarette smoke inhibits the NLRP3 inflammasome and leads to caspase-1 activation via the TLR4-TRIF-caspase-8 axis in human macrophages. FASEB J (2020) 34(1):1819–32. doi: 10.1096/fj.201901239R 31914643

