# Supplementary material for: Multiple TLRs elicit alternative NLRP3 inflammasome activation in primary human monocytes independent of RIPK1 kinase activity
Source: Front Immunol. 2023 Oct 26;14:1092799. doi: 10.3389/fimmu.2023.1092799 (PMC10639122; doi:10.3389/fimmu.2023.1092799)
Supplement: Supplementary file 1 [file DataSheet_1.pdf]

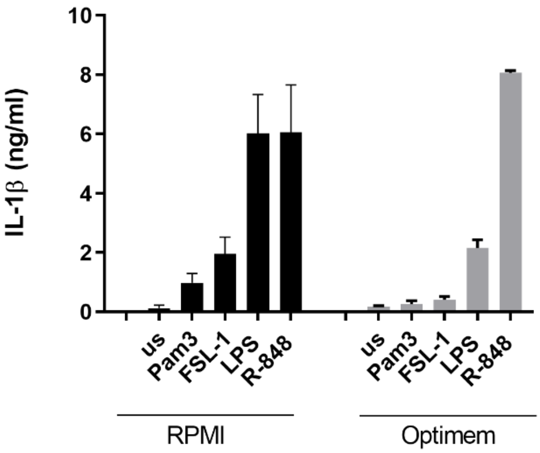

Supplementary Figure S1. IL-1β secretion is reduced in monocytes stimulated in Optimem. Primary human monocytes were unstimulated (us) or stimulated for 24h with 100ng/ml Pam3, 1ng/ml FSL-1, 10ng/ml LPS or 2µg/ml R-848 in RPMI media with 5%FBS or Optimem. IL-1β secretion was measured by ELISA and shown from the same donor as the mean±SD of technical triplicates.

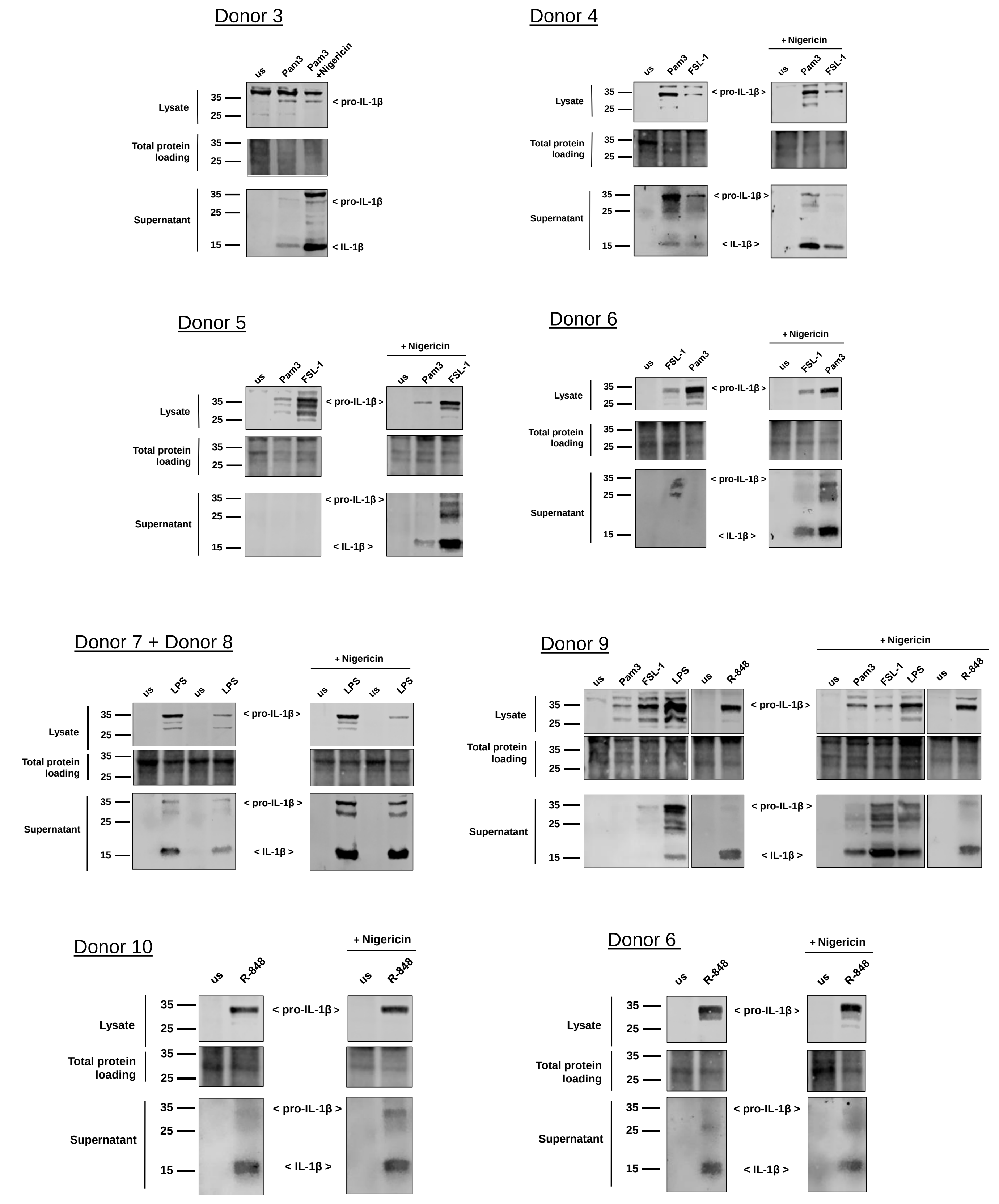

Supplementary Figure S2. Monocytes release mature IL-1β into the cell supernatant following TLR1/2, 2/6, 4 and 7/8 activation. Primary human monocytes were unstimulated (us) or stimulated for 24h with 100ng/ml Pam3, 1ng/ml FSL-1, 10ng/ml LPS or 2µg/ml R-848 in the absence or the presence of 10µM nigericin. Western blot analysis was performed for pro-IL-1β (31kDa) and mature IL-1β (17kDa) as well as total protein loading. Each set of western blots is from an independent donor, apart from the middle left panel (Donor 7 +8), where 2 individual donors have been run on the same gel.

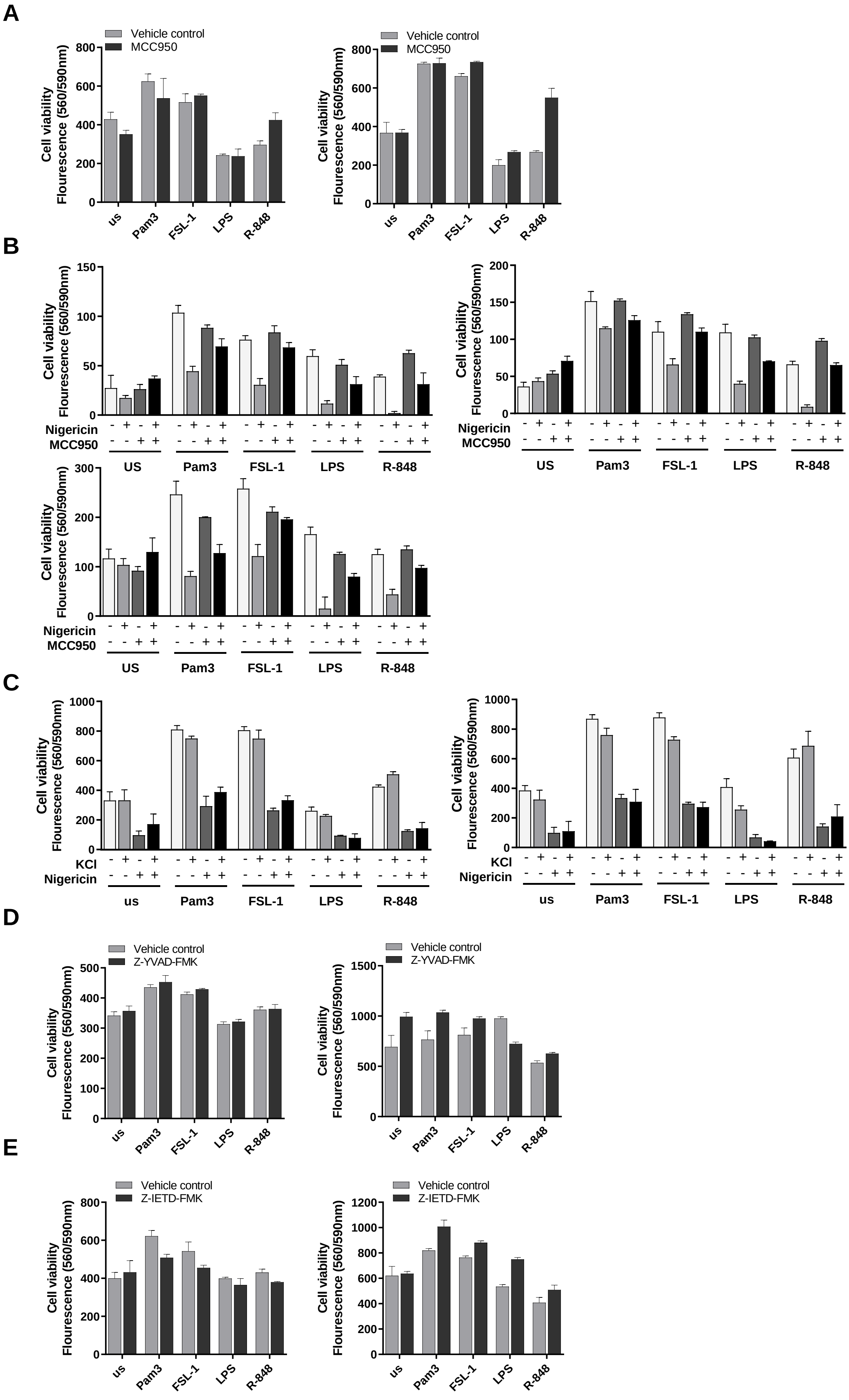

Supplementary Figure S3

F

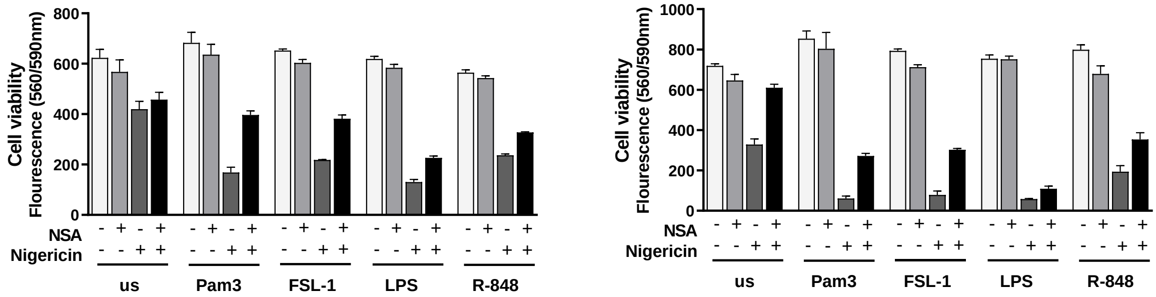

Supplementary Figure S3. Cell Viability assays. Monocytes were unstimulated (us) or stimulated for 24h with 100ng/ml Pam3, 1ng/ml FSL-1, 10ng/ml LPS or 2µg/ml R-848 in the absence or the presence of (**A**) MCC950, (**B**) MCC950 and 10µM nigericin, (**C**) KCl and nigericin, (**D**) Z-YVAD-FMK, or (**E**) Z-IETD-FMK. (**F**) Monocytes were unstimulated (us) or stimulated for 5.5h with 100ng/ml Pam3, 1ng/ml FSL-1, 10ng/ml LPS or 2µg/ml R-848 in the absence or presence of 10µM necrosulfonamide (NSA) and/or 10µM nigericin. Cell viability was determined by cell titer blue assay and shown from two independent donors as the mean±SD of technical triplicates.

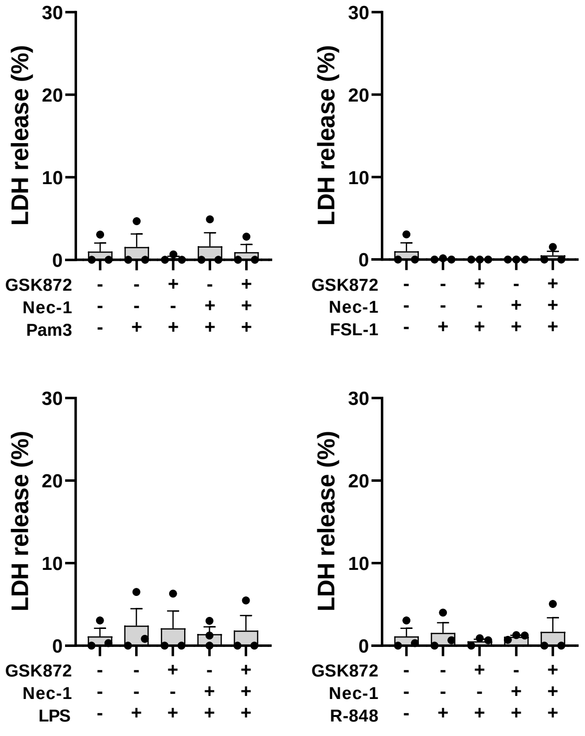

Supplementary Figure S4. RIPK1 inhibition did not induce cell death in primary human monocytes. Cells were unstimulated or stimulated with 100ng/ml Pam3, 1ng/ml FSL-1, 10ng/ml LPS or 2µg/ml R-848 in the presence of 40µM necrostatin-1 (Nec-1) or 2.5 µM GSK872. Cell death was measured by a lactate dehydrogenase (LDH) release assay. Data are displayed from 3 individual donors and displayed as the mean±SEM.

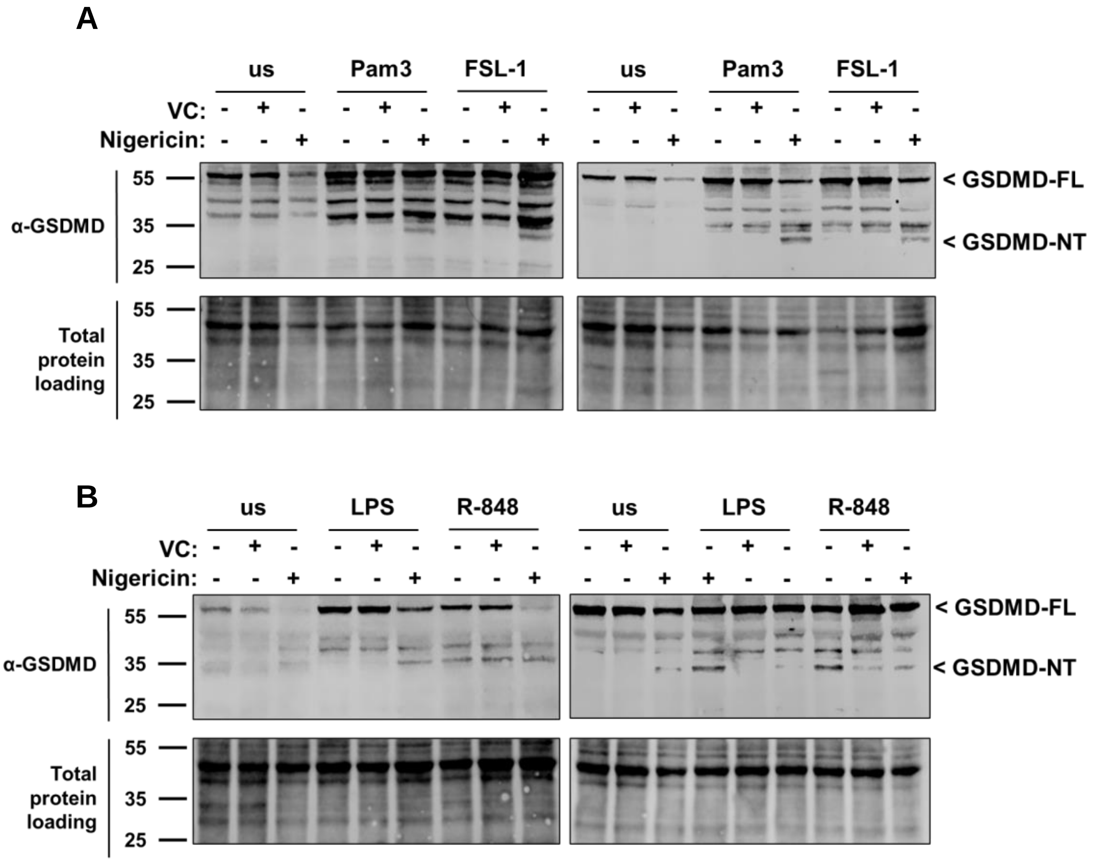

Supplementary Figure S5. GSDMD is cleaved following activation of R-848 in the absence of nigericin. Monocytes were unstimulated (us) or stimulated for 24h with (**A**) 100ng/ml Pam3, 1ng/ml FSL-1, (**B**) 10ng/ml LPS or 2µg/ml R-848, in the presence of 10µM nigericin or a vehicle control (VC). Gasdermin D (GSDMD-FL, 53kDa), gasdermin N-terminus (GSDMD-NT, 31kDa) and total protein loading were assessed in two independent donors by western blot.

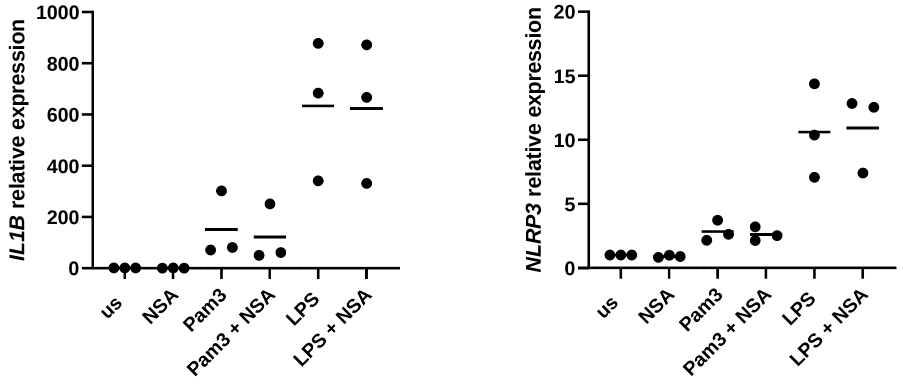

Supplementary Figure S6. Necrosulfonamide (NSA) does not affect *IL1B* and *NLRP3* expression. Monocytes were unstimulated (us) or stimulated for 5.5h with 100ng/ml Pam3 or 10ng/ml LPS and NSA was added for the last 2h. The expression of *IL1B* and *NLRP3 were* normalized to the geometric mean of the reference genes *GAPDH* and *HPRT1*. Data are displayed from 3 individual donors showing the mean relative expression.
